# Terminologies for text-mining; an experiment in the lipoprotein metabolism domain

**DOI:** 10.1186/1471-2105-9-S4-S2

**Published:** 2008-04-25

**Authors:** Dimitra Alexopoulou, Thomas Wächter, Laura Pickersgill, Cecilia Eyre, Michael Schroeder

**Affiliations:** 1 Biotechnology Center (BIOTEC), Technische Universität Dresden, Dresden, D-01062, Germany; 2 Unilever Corporate Research, Colworth, MK44 1LQ, UK; 3 Unilever – Safety and Environmental Assurance Centre, Colworth, MK44 1LQ, UK

## Abstract

**Background:**

The engineering of ontologies, especially with a view to a text-mining use, is still a new research field. There does not yet exist a well-defined theory and technology for ontology construction. Many of the ontology design steps remain manual and are based on personal experience and intuition. However, there exist a few efforts on automatic construction of ontologies in the form of extracted lists of terms and relations between them.

**Results:**

We share experience acquired during the manual development of a lipoprotein metabolism ontology (LMO) to be used for text-mining. We compare the manually created ontology terms with the automatically derived terminology from four different automatic term recognition (ATR) methods. The top 50 predicted terms contain up to 89% relevant terms. For the top 1000 terms the best method still generates 51% relevant terms. In a corpus of 3066 documents 53% of LMO terms are contained and 38% can be generated with one of the methods.

**Conclusions:**

Given high precision, automatic methods can help decrease development time and provide significant support for the identification of domain-specific vocabulary. The coverage of the domain vocabulary depends strongly on the underlying documents. Ontology development for text mining should be performed in a semi-automatic way; taking ATR results as input and following the guidelines we described.

**Availability:**

The TFIDF term recognition is available as Web Service, described at

## Background

The engineering of ontologies is still a new research field. There does not yet exist a well-defined theory and technology for ontology construction. This means that many of the ontology design steps remain manual and a kind of “art” and intuition [1-3]. There exists a variety of different ontologies, constructed for different purposes and projects.

As far as the biomedical ontologies are concerned, during the last years there have been major efforts in the biological community for organizing biological concepts in the form of controlled terminologies or ontologies [4-7]. A key difference between terminologies and ontologies is that the former lack the semantic depth of the latter. However, when it comes to design, terminologies can serve as basis for ontologies and vice-versa. An example where a terminology can serve for ontology is that of the Gene Ontology [6], which provides a controlled vocabulary to describe gene and gene products in any organism. On the other side, the Gene Ontology Next Generation (GONG) project [8] aims at the migration of current bio-ontologies to a richer and more rigorous status, using formal representation languages like OWL. Examples of true ontologies are the GALEN project [9] and the Systematized Nomenclature of Medicine (SNOMED) [10] which are based on Description Logic for concept representation and the Foundational Model of Anatomy (FMA) [11] which is based on frames representing information about anatomical classes, designed so that content can be maintained as a dynamic resource and can be used as terminologies.

There have also been developed systems to provide interoperability among different ontologies, such as the Unified Medical Language System [12] in order to provide a common frame of reference among the different research communities. The Open Biomedical Ontologies (OBO) Foundry [13] hosts over 60 open source ontologies associated with phenotypic and biomedical information, such as the Mouse Anatomy (MA) [7] and the Cell Ontology (CL) [14]. Bodenreider and Stevens [15], Blake and Bult [16] and Baker *et al*. [17] give overview on biomedical ontologies, the consortia involved, formalisms as well as semantic web technologies and representation tools.

Semantic meta-information provided in the form of ontologies has proven useful in order to search [18,19] or index large collections of documents (e.g. MeSH for indexing MEDLINE [20]). Meta-information found for text documents is often general (keyword list) or still too complex for an automated evaluation (article abstract). Finding terms of controlled vocabularies in text overcomes this shortage, while relations between terms provide the necessary navigation structures.

Ontological background knowledge can serve to answer questions with knowledge-based search engines, by easing the task of finding relevant documents through the term automatic annotation [18,21,22]. In the domain of lipoprotein metabolism, for example, a search for “analphalipoproteinemia” will retrieve articles for Tangier's disease, which is actually a synonym. In case of a syndrome, such as the “metabolic syndrome”, in a properly designed ontology the articles retrieved will contain symptoms and other characteristics for it (e.g. type II diabetes, hypertension, insulin resistant, low HDL, hypertension, all of them being parts of the metabolic syndrome). Researchers explore literature on different parameters that can affect the lipoprotein metabolism, such as the phenotype, genotype and age of the patients/animals tested, environmental factors and lifestyle, specific lipoprotein and enzyme concentrations and others. Questions like ‘what is the activity of cholesterol ester transfer protein in diabetes’, ‘which cells/tissues is apoE expressed in’, ‘what is the impact of a fish oil diet on metabolic syndrome individuals’, ‘which genes/proteins/metabolites are hypertension-specific’ can be answered with the use of a well designed ontology on lipoprotein metabolism, containing terminology found in literature with semantically interconnected terms.

The GoPubMed search engine [18,19] allows users to explore PubMed search results with the Gene Ontology (GO) [6] and Medical Subject Headings (MeSH) [20]. GoPubMed retrieves PubMed abstracts for a search query, detects terms from the GO and MeSH in the abstracts, displays the subset of GO and MeSH relevant to the keywords and allows for browsing the ontologies and displaying only articles containing specific GO and MeSH terms. The search engine is developed in a way that any ontology (e.g. a Lipoprotein Metabolism Ontology) can be easily integrated and used for a domain-specific literature search. One of the benefits of such an ontology-based literature search is the categorization of abstracts according to a specific ontology, allowing users to quickly navigate through the abstracts by category and providing an overview of the literature. It can also automatically show general ontology terms related to the original query, which often do not even appear directly in the abstract.

In this paper, we introduce design principles for ontologies used for text mining, based on our personal experience with the manual development of a Lipoprotein Metabolism Ontology. A key problem in this context is the generation of terms, which is corroborated by Castro *et al*. [3], who compared different ontology design methods and tools all of which lacked automated term recognition. The paper is organized as follows. We first introduce the design principles followed when designing the lipid metabolism ontology and turn to the question how to automate the generation of terms. We introduce two methods to identify terms and evaluate them together with two existing tools for this task.

## Methods

### Ontology design principles

The Open Biomedical Ontologies (OBO) Foundry provides ontology design principles concerning the syntax, unique identifiers, content and documentation of ontologies to be added or edited, as a common agreement between users/editors. OBO principles that are not discussed later (but were followed during the Lipoprotein Metabolism Ontology design) refer mainly to the use of a common shared syntax (OBO syntax and extensions or OWL), the insertion of a unique identifier per term, the relations included in the OBO Relation Ontology and the clearly delineated content (terms in different ontologies should provide distinguishable descriptions of a concept). The success of the OBO representation format is attributed to its informal expressivity, combined with the ability of conversion into OWL and vice-versa.

The only OBO principles we did not follow were the free availability and collaboration with other OBO Foundry members, due to corporate reasons. However, we present the knowledge acquired and the problems faced during the ontology design. The following guidelines, as well as the decisions, compromises and problems described later derive from our experience during the manual development of the Lipoprotein Metabolism Ontology.

Some basic steps that should be followed during the design of any ontology include *identifying the range of intended users*, *deciding on the purpose and main research area* of the ontology and defining/predicting further *possible applications* (e.g. GO has also been used by the search engine GoPubMed [18,19] and by GoMiner [23] for gene expression data evaluation, although its initial purpose did not include use for text-mining). Important points to start from are *literature scanning* for deciding on the basic concepts as well as *the insertion of a textual definition for each term*. *Formulation of questions* is also crucial [24]. Examples of questions that researchers from Unilever needed to answer were: “what is the activity of cholesterol ester transfer protein (CETP) in diabetes?”, “which tissues is apoE expressed in?”, “what is the impact of fish oil diet in metabolic syndrome patients?”, etc, indicating that terms such as ‘CETP’, ‘diabetes’, ‘apoE’, ‘diet’, ‘fish oil diet’, ‘metabolic syndrome’ and ‘patient’ should be included in the ontology. *Reusing existing ontologies* that may cover to some extent the ontology under design or could be inserted as a separate branch of the ontology is also a possibility. In the case of the Lipoprotein Metabolism Ontology (LMO), we needed to include information on diet. For this purpose, we included the Nutrition Ontology from the NCI Cancer Nutrition Ontology Project [25] as a separate part under diet. *Deciding on a label for each concept* is one of the most crucial steps during the structuring of the ontology. This task is difficult for humans as it requires good knowledge of the domain of interest so as to group concepts on the hierarchy in a semantically meaningful way. It is even more difficult for machines to do this automatically. There has been previous work on automatic labeling of document clusters [26] by using the most frequent and most predictive words in clusters of documents, but there is still work to be done on that. One must firstly concentrate on the semantics of a term, decide what is really needed to be expressed with that term and then choose the appropriate name. Last, but not least, and perhaps one of the very first steps of the designing procedure is the selection of a suitable *ontology editor*. We used the Protégé OWL plug-in [27] for building the ontology and CmapTools [28] for visualization. Ontology visualization is crucial when the knowledge engineer and the domain expert are two different persons and need to agree on the different versions of the ontology.

**Figure 1:**
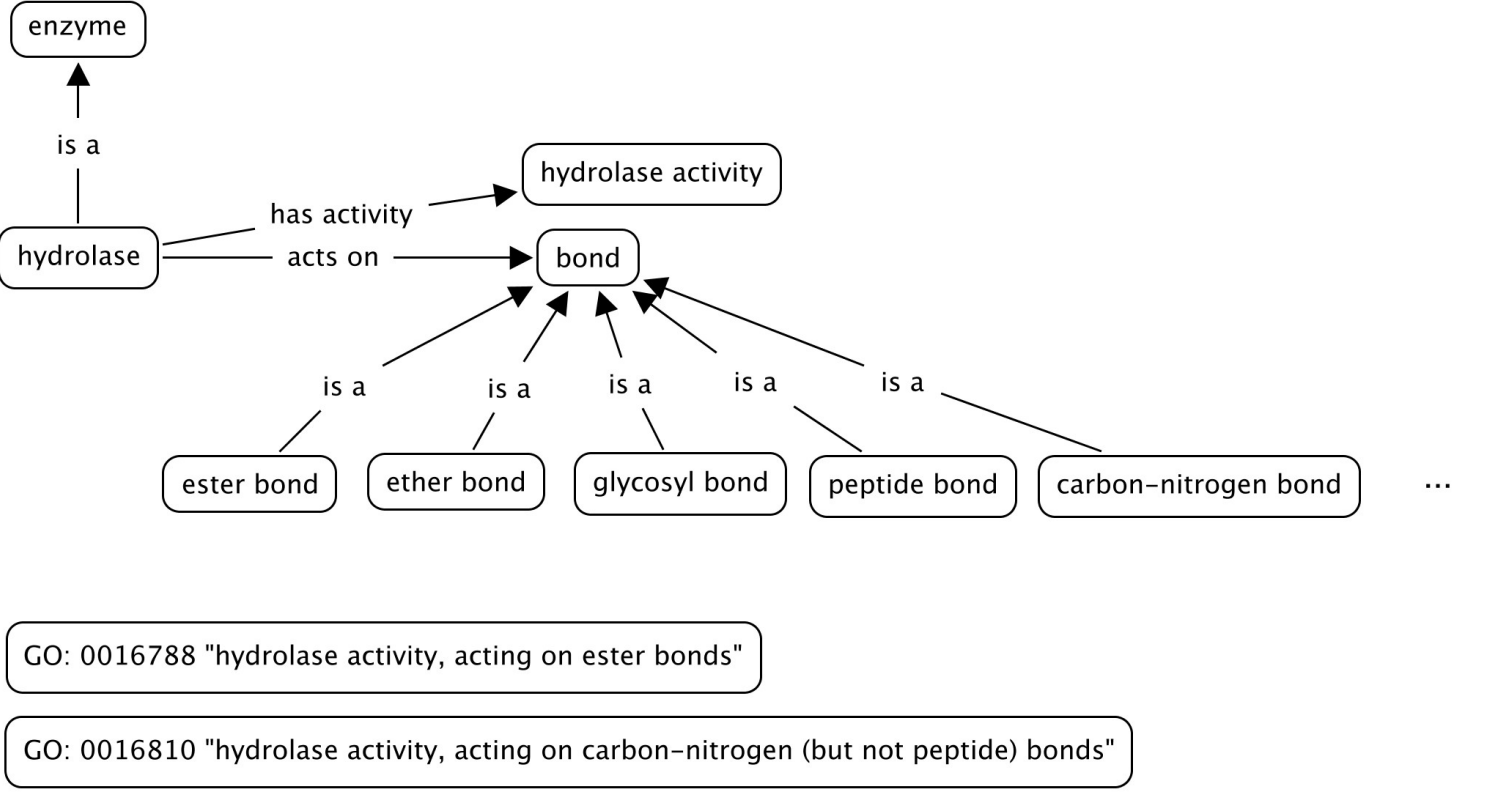
**Problematic terms – the hydrolase activity example**. Terms like hydrolase, hydrolase activity, bond, ester bond and relations between them (e.g. acts on) can be easily found in text, whereas full GO terms such as ‘hydrolase activity, acting on ester bonds’ are unlikely to appear literally in an article.

With GO we experienced some limitations for text-mining. For example, it is unlikely that a descriptive label such as ‘cell wall (sensu Gram-negative bacteria)’ will literally appear in text. A comprehensive overview of such problems is provided by Smith *et al*. [29]. There often exist ontology terms that are unlikely to appear as such in text but are rather of a structuring nature. For example, the terms ‘hydrolase activity, acting on ester bonds’ (GO:0016788) or ‘hydrolase activity, acting on carbon-nitrogen (but not peptide) bonds’ (GO:0016810) include several different types of information: activity (hydrolase), type of bond affected (ester or carbon-nitrogen) and exception (but not peptide) (see Figure 1). These should be 3 different branches of the tree, combined with relations, therefore structuring ‘logical formulas’. For example, in the case of the second term (GO:0016810), the exception could be expressed as a certain condition: the protein has a hydrolase activity and is acting on carbon nitrogen bonds, but *not in all* bonds (peptide bonds are excluded). Aranguren *et al*. [30] provide a simple and indicative example of the problem: a Person is a Man or a Woman, a Man has Testis, a Woman has no Testis, but what happens in the case of a Eunuch (who is actually a man without Testis)? There is a need for distinguishing between relations that are strict “always” rules and “normally” or “usually” relations that can also allow for exceptions. Biomedical terms are usually connected with “usually” relations between them. Another example is the definition of mammals: a simple definition [31] can be ‘warm-blooded vertebrate animals belonging to the class mammalia, including all that possess hair and suckle their young’. Therefore, one can say that all mammals give birth to and suckle their young. But there exists the exception of the monotremes, which are mammals that lay eggs instead of bearing live young. The definition here would be “mammals are animals that *normally* bear live young and suckle them” and the exception “monotremes are mammals that lay eggs”. Another example is given by Hoehndorf *et al*. [32] (from the Foundational Model of Anatomy), where “*every instance of a human body has as part an appendix*”, corresponding to an idealized (canonical) “normal” human. However, an individual human body may lack an appendix as a part, demonstrating that canonical ontologies do not always represent default knowledge and should include exceptions. Hoehndorf *et al*. developed a methodology for representing canonical domain ontologies within the OBO foundry by adding an extension to the semantics for relationships in the biomedical ontologies that allows for treating canonical information as default. Rector [33] explored some of the alternatives in OWL and related languages for dealing with issues such as exceptions (predictable and not) and limited expressivity. Rector's analysis is divided in four cases, which can be resolved with OWL, more precise logical formulation (OWL-DL), more explicit context and generalized common information and other more complicated methods.

Compositional structure of terms is a major bottleneck for ontology design, especially when it comes to text mining, as the relations between terms must be as simple as possible. Ogren *et al*. [34,35] have performed an analysis of the term names in the GO to investigate substring relations between terms and revealed that 65.3% of all GO terms contain another GO term as a proper substring. These terms can be categorized into two groups: GO terms that contain other GO terms as proper substrings (e.g. ‘hydrolase activity, acting on acid sulfur-sulfur bonds’ (GO: 0016828) and ‘hydrolase activity’ (GO: 0016787)) and GO terms that contain strings that seem to recur frequently (e.g. ‘regulation of’ in GO, ‘predominance of’ in the Lipoprotein Metabolism Ontology).

Text-mining ontologies can be extensions of annotation ontologies which enrich annotation ontologies with synonyms suitable for text-mining. Some decisions and compromises have to be made on the relationships and on the labels defined during the concept hierarchy design.

#### Decisions that need to be made during the ontology design

*Keep or dismiss a term*: When using the ontology for text-mining over a specific biomedical domain, it is important to include terms specific enough to define the domain and also general enough to cover it entirely. For example, including information on ‘kinetics’ during the design of the Lipoprotein Metabolism Ontology is crucial. But ‘kinetics’ is too general as a term, as the distinction between different kinds of kinetics is important (e.g. when querying PubMed for ‘kinetics’, there are retrieved articles referring to ‘kinetics of phenols’ or a ‘reconstruction kinetics well’, irrelevant to the domain of interest). On the other hand, the term ‘lipoprotein kinetics’ is too specific and documents mentioning it do not cover all essentials known in lipoprotein kinetics. Searches for “lipoprotein kinetics”, “lipoprotein” and “kinetics” and retrieval of relevant articles (e.g. PMID: 12606523 ‘Differential regulation of lipoprotein kinetics by atorvastatin and fenofibrate in subjects with the metabolic syndrome.’) lead to the decision that the best term to use for ‘lipoprotein kinetics’ is the exact term. There already exist previous efforts on automatic labeling of document clusters and identification of ontology components, based on Natural Language Processing techniques or hierarchical and suffix-tree clustering [36,37,26].

*Decide on ontology design/relations*: the ontology must be a subsumption ontology. It can be either a structured vocabulary/terminology containing only child-parent relationships (mostly ‘is_a’ and ‘part_of’) between concepts or an ontology of different complexity that could be easily translated into a simple hierarchy. It should also be rich in synonyms and textual definitions (mentioned earlier), that would be useful for disambiguation. For the Lipoprotein Metabolism Ontology we used the Protégé OWL plug-in, with concepts being the term labels (e.g. human / Tangier's disease) and instances the term synonyms/variants (e.g. patient, test person, experimentee / analphalipoproteinemia).

*Decide on synonyms*: researchers do not have strict and formal ontologies or nomenclatures in their minds when composing a scientific article and therefore use terminology of differing granularity. They often use parent terms to refer to a child term, or vice-versa (e.g. ‘coronary artery disease (CHD, CAD)’ is child of ‘cardiovascular disease’, but in many cases authors are treating them the same). Again literature scanning, for both child and parent term, will help to clarify how researchers refer to different terms. Another problematic case is that of the different lipoprotein subclasses (based on particle size, buoyant density, composition, etc.) where there do not exist clear limits between them. Depending on the way of measurement and the difference in surface lipid content, they can be expressed in different ways. For example, in the case of LDL, there are 5 different subclasses based on particle size (LDL I-V), but there are also references such as ‘small dense LDL’ or ‘buoyant LDL’ that are very often found in text but could contain a mixture of different subclasses. Since we need to keep only a simple hierarchy with parent-child relationships, we do not incorporate any “definitional” information (e.g. that ‘small dense LDL’ consists of a mixture of LDLIII and LDLIV). In these cases, we put the synonyms according to the authors’ use, for example ‘small dense LDL’ as a synonym for LDL III and ‘buoyant LDL’ or ‘large LDL’ as synonyms for LDL I [38]. A similar example from the GO is that of ‘transporters and carriers’. In every day language ‘transporter and carrier’ is the same as ‘transporters or carriers’, but logically they are different.

*Handle term variation*: terms like ‘Tangier disease’, ‘Tangier's disease’ or ‘Tangiers disease’ are variants of the same term. Terms like ‘LDL I’, ‘LDL-I’, ‘LDL-1’, ‘LDL 1’, ‘LDL1’ and ‘LDLI’ are also variants of the same term. The process of manually inserting such lexical variants (with hyphens, apostrophes, slashes, or even American/British spelling variants) in the ontology is tedious and time-consuming. There exist programs that handle such variations by producing the normalized form of a term, such as the UMLS Lexical Variant Generation Program [39]. For the Lipoprotein Metabolism Ontology we did not use such a variant generation program. We included only term variants we could find in literature.

#### Compromises that need to be made, problems, inconsistencies

There must be made some compromises to retain a correct ontology (meaning that it contains valid relations) and still get the best possible results from text-mining:

*Ambiguity* resulting either from identical *abbreviations* for different terms (e.g. ‘CAM’ can stand for ‘constitutively active mutants’, ‘cell adhesion molecule’, or ‘complementary alternative medicine’), or *ambiguous term labels* (e.g. ‘embryo’ for ‘mouse embryo’ or ‘male’ for ‘male patients’) is always a problem. Abbreviations and acronyms should be included in the ontology, but conservatively or with an appropriate algorithm that could handle them. Word sense disambiguation is a salient point here; knowledge sources like long-form/short-form combinations, domain (context under which the word is used) and collocations (adjacent words/terms) can be exploited to provide the correct sense of the term [40]. For the case of incomplete term labels, let us consider the following example: we are only interested in experiments performed in human patients and need to distinguish between human- and animal- referring articles. One option is to insert into the ontology only human-specific terms, such as ‘experimentee’, ‘patient’, ‘man’, ‘boy’, etc. ‘Male’ cannot be in the ontology, since it could also be referring to animals. Another option is to maintain a list of human- and animal- specific words or expressions and then transform the algorithm in a way that one could make a Boolean selection (e.g. AND human, NOT animal) in the query and finally include or exclude the results for the specific selections.

*Try to avoid any possible inconsistencies*. To illustrate the implication of inconsistencies on reasoning, let us describe the following example: a researcher is interested in the different lipoprotein levels in patients of different race and geographical location, since there has been evidence that these two factors affect lipoprotein metabolism. Combination of geographical information as well as racial information in one part of the ontology is, therefore, needed. Many articles refer to “African-Americans” as “blacks”, so the term must be included under ‘ethnic group’. Then the following must be valid: define ‘Caucasian’, ‘African’ and ‘Asian’ as ‘ethnic group’, ‘American’ is a ‘Caucasian’, ‘African-American’ is a ‘African’, ‘African-American’ is a ‘American’, ‘African-American’ is ‘black’ (synonym), ‘Caucasian’ is white (synonym) but ‘African-American’ cannot be ‘Caucasian’ or ‘white’ (although he is ‘American’). This is similar to the case of mammals that lay eggs or the ‘Man, Woman, Eunuch’ example described earlier; people very often formulate rules such as “normally is-a”, as there are always exceptions. For the LMO we excluded the ‘American’ concept and added ‘African-American’ as child of ‘African’ and ‘Hispanic-American’ as child of ‘Caucasian’.

## Results

The Lipoprotein Metabolism Ontology (LMO) was manually built in collaboration with domain experts from Unilever for the purpose of document retrieval. It consists of 223 concepts and 623 additional synonyms, with an average term length of 14 (2 words of 7 characters). A concept as used here consists of a concept label and optional synonymous terms. A term can be any word or phrase of relevance to the studied domain. Together with the Nutrition Ontology from the NCI Cancer Nutrition Ontology Project [25], the LMO contains in total 522 concepts and 964 additional synonyms, with an average term length of 15 (2 words of 7.5 characters). Concerning the relations between the concepts, the mean number of parents is 2 (with a maximum of 3) and the mean number of siblings is 5 (with a maximum of 10). We did not include the Nutrition Ontology terms in the experiment, as we only wanted to compare the terminology created manually by us with the automatically derived terminologies from the different term extraction methods. For Automatic Term Recognition (ATR), a ‘lipoprotein metabolism’-specific corpus was created, consisting of 300 abstracts collected from PubMed with the query “lipoprotein metabolism” (limit for Review papers). These 300 abstracts were the maximal number of articles where all methods delivered results. Five different ATR methods were tested on that corpus, namely Text2Onto, OntoLearn, Termine [41-43] and two methods developed in-house, one considering the relative frequency (RelFreq) of a term in the corpus and the other (TFIDF) additionally using the document frequency derived from all phrases contained in NCBI's PubMed database. Termine [43] considers several statistical characteristics of the candidate term, such as the total frequency of occurrence in the corpus, the frequence of the term as part of other longer candidate terms (and the number of these) and the length of the candidate term (in number of words). Text2Onto [41] is based on algorithms calculating the Relative Term Frequency and TFIDF, as well as Entropy and the C-value/NC-value used by Termine in order to extract the concepts. It further exploits the hypernym structure of WordNet [44], matches Hearst patterns [45] and others in the corpus in order to get the relations (subclass_of, part_of, instance_of), but at this point we only examined the terminology extraction precision. OntoLearn [42] uses a linguistic processor and a syntactic parser in order to extract a list of syntactically plausible terminological noun phrases. For filtering “true” terminology, OntoLearn is based on two measures, namely Domain Relevance and Domain Consensus, which calculate the specificity of a candidate term with respect to the target domain via comparative analysis across different domains as well as the distributed use of a term in a domain. OntoLearn was excluded from further analysis, as it only generated a few terms so that a meaningful comparison would be possible, see Table 1. Text2Onto was only included in the analysis for 300 abstracts as it was not possible to process all 3066 review article abstracts for “lipoprotein metabolism” listed in PubMed. We performed a bipartite analysis. We tried to automatically reconstruct the manually created LMO terminology, compared the terms predicted by the four methods to the current LMO terms and also evaluated manually the top 1000 retrieved terms. All automatic comparisons between candidate terms and LMO were not case sensitive.

**Table 1:** Top 25 predicted terms per method. Listing of the top 25 predictions for TFIDF, RelFreq, Termine, Text2Onto and OntoLearn. Terms relevant to the lipoprotein metabolism domain are marked with x.

	**Methods**								
**Rank**		**TFIDF**		**RelFreq**		**Termine**		**Text2Onto**	**OntoLearn**

**1**	x	metabolic syndrome		review	x	low-density lipoprotein	x	patient	Mutation
**2**	x	HDL	x	metabolic syndrome	x	cardiovascular disease	x	disease	fish oil
**3**	x	atherosclerosis	x	diabetes	x	metabolic syndrome		risk	hypercholesterolaemia
**4**		review	x	atherosclerosis	x	risk factor		effect	Serum
**5**	x	LDL	x	HDL	x	cardiovascular risk		study	progression of atherosclerosis
**6**	x	cardiovascular disease	x	LDL	x	high-density lipoprotein		level	Apheresis
**7**	x	diabetes	x	cardiovascular disease	x	low-density lipoprotein cholesterol	x	atherosclerosis	omega-3
**8**	x	dyslipidemia	x	cholesterol	x	high-density lipoprotein cholesterol	x	cholesterol	treatment of hypertriglyceridemia
**9**	x	high-density lipoprotein		type	x	fatty acid	x	lipoprotein	reductase inhibitor
**10**	x	cholesterol		article	x	coronary heart disease	x	statin	Triglyceride
**11**	x	low-density lipoprotein	x	fatty acids	x	coronary artery disease		role	adhesion molecule
**12**	x	cardiovascular risk	x	high-density lipoprotein		clinical trial		syndrome	Evolution
**13**	x	fatty acids		role	x	ldl cholesterol	x	diabetes	purification process
**14**		article	x	dyslipidemia	x	heart disease	x	trial	Prescription omega-3
**15**	x	insulin resistance	x	low-density lipoprotein	x	diabetes mellitus		protein	omega-6
**16**		type	x	cardiovascular risk	x	omega-3 fatty acid	x	risk factor	hiv-infected
**17**	x	statin	x	hypertension		blood pressure	x	treatment	marker of inflammation
**18**	x	hypertension		combination	x	oxidative stress		event	strong evidence
**19**	x	inflammation	x	insulin resistance		increased risk		therapy	attractive target
**20**	x	VLDL		protein		density lipoprotein		review	accelerated atherosclerosis
**21**	x	lipid metabolism	x	disease	x	cardiovascular risk factor		type	internalization
**22**		combination		studies		coronary artery		mechanism	Scenario
**23**		role	x	inflammation	x	statin therapy		evidence	protease inhibitor
**24**	x	oxidative stress		association	x	plant sterol		development	inflammatory cell
**25**	x	obesity	x	plasma	x	reverse cholesterol transport		use	inflammatory marker

### Reconstruction of LMO terminology

Consider Table 2, which shows the percentage of terms that can be generated by the four methods. The first table lists the results for LMO alone, the second for LMO and terms considered relevant after manual inspection. Furthermore, we distinguish precision and average precision. The latter takes the ranking of terms into account:

(1) $average\;precision=\frac{\sum_{r=1}^{N}\left(P\left(r\right)\times rel\left(r\right)\right)}{number\;of\;retrieved\;terms}$, with

(2) $rel\left(r\right)=-\frac{2}{N^{2}}\left(r-1\right)+\frac{2}{N}$

**Table 2:** Precision and Average Precision (rank dependent) for top 50 / 200 / 1000 predictions for 4 methods (TFIDF, Relative Frequency, Termine, Text2Onto) in terms of coverage of LMO and relevant vocabulary. The key finding is that among the top 1000 predictions there are up to 51% terms, which are in the LMO or considered good terms by expert, implying that automated term recognition can play an important role in semi-automated ontology design.

	**LMO**							
	**Precision**				**AveragePrecision**			

**Top**	**TFIDF**	**Termine**	**Text2Onto**	**RelFreq**	**TFIDF**	**Termine**	**Text2Onto**	**RelFreq**

**50**	**35%**	19%	17%	**35%**	**65%**	54%	38%	54%
**200**	20%	10%	12%	**22%**	**42%**	28%	23%	37%
**1000**	**8%**	4%	5%	**8%**	**21%**	12%	12%	20%

	**LMO + Domain expert**							

	**Precision**				**Average Precision**			

**Top**	**TFIDF**	**Termine**	**Text2Onto**	**RelFreq**	**TFIDF**	**Termine**	**Text2Onto**	**RelFreq**

**50**	**75%**	67%	33%	56%	86%	**89%**	52%	70%
**200**	**55%**	40%	49%	49%	**74%**	65%	38%	60%
**1000**	**29%**	20%	14%	28%	**51%**	40%	25%	45%

where *r* is the rank of retrieval and *P(r)* is the precision at a cut-off rank. For each of the four methods we list the percentage of relevant terms for the top 50, top 200, and top 1000 predictions. The results show that the precision for the top 50 predictions for LMO ranges from 17-35% and 4-8% for the top 1000 predictions. Using LMO and the expert terms leads to better results of up to 75% for the top 50 predictions and up to 29% for the top 1000. Considering the average precision and thus the ranking of terms, results for the top 50 predictions go up to 89% and for the top 1000 up to 51%. Generally, Termine which favours long terms performs well for the top 50, because long terms are a good indicator of a relevant term. However, there are many short terms, which are relevant, too. The TFIDF and RelFreq methods can pick up these terms, as they include background knowledge, i.e. frequencies of terms in PubMed. By and large, Text2Onto does not perform so well as it neither includes domain-specific background knowledge (as in the case of the TFIDF developed in-house) nor the ranking pursued by Termine, which is biased towards longer frequent terms. Text2Onto suggested short and very general terms, like ‘use’, ‘effect’, ‘study’, ‘event’, etc. Although we explicitly deactivated the relation extraction part for this experiment, it is not clear why Text2Onto persisted in ranking these terms in the top of the list. Overall, the results are encouraging, as they indicate that a large part of the terminology can be generated automatically.

**Figure 2:**
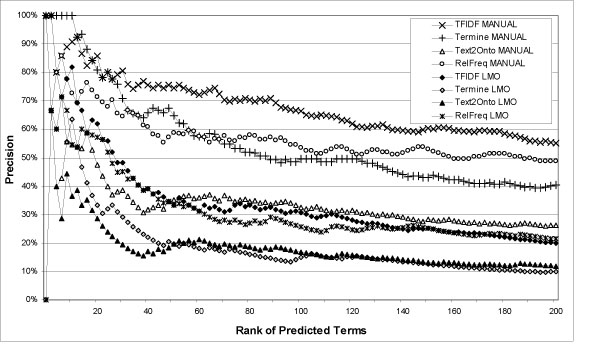
**Overlap with manually curated LMO and manual evaluation**. Precision at a certain rank *r* represents each method's capability to recognize domain relevant terms within the top *r* retrieved terms. The chart shows the overlap within the top *r* predicted terms with LMO and the manual evaluation (MANUAL). For example, from the top 50 predicted terms by Text2Onto, 20% are in LMO and 36% are correct according to the manual evaluation.

**Figure 3:**
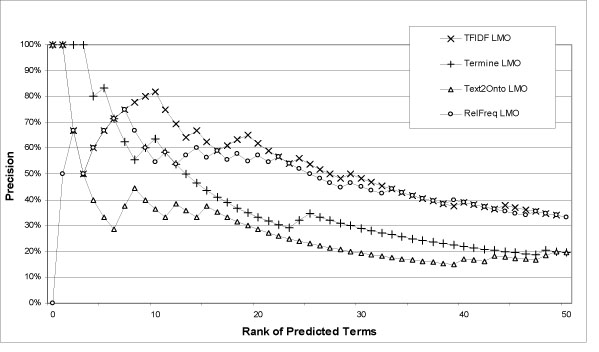
**Overlap with LMO.** Precision at a certain rank *r* represents each method's capability to recognize domain relevant terms within the top *r* retrieved terms. The chart shows the overlap within the top *r* predicted terms with LMO. For example, from the top 20 predicted terms by TFIDF, 65% are in LMO.

**Figure 4:**
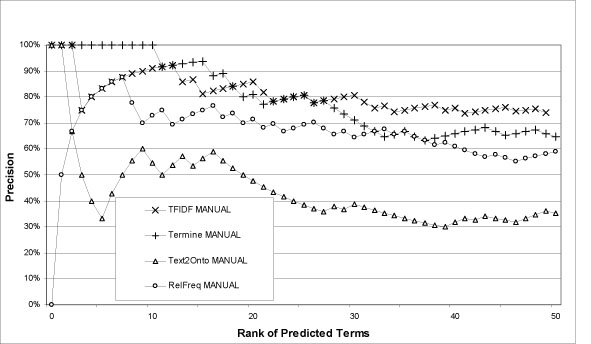
**Overlap with controlled lipoprotein metabolism vocabulary and additional manual evaluation (makes sense/makes no sense)**. Precision at a certain rank *r* represents each method's capability to recognize domain relevant terms within the top *r* retrieved terms. The chart shows the overlap within the top *r* predicted terms with the manual evaluation. For example, from the top 10 predicted terms by Termine, 100% are relevant to lipoprotein metabolism.

Concerning recall, consider Table 3. 3066 documents contain only 53% of the LMO terms literally. TFIDF manages to predict up 39%, which is an encouraging result. Increasing the document base to 50.000 only 71% of the LMO terms are included indicating a possible upper limit. Figure 2 provides an overview of the results we acquired from these comparisons. Figures 3 and 4 provide zoom-ins of Figure 2, describing the performance of each method in the top 50 predicted terms.

**Table 3:** Coverage of LMO terminology in selected document sets. The table sets the upper limit of terms that can be found with text-mining: Even a large text base with 50,000 documents contains only 71% of LMO terms. TFIDF can predict up to 38% of LMO terms.

	***LMO terminology predicted by TFIDF***		***LMO terminology literally contained***	

	**1000**	**all**	

300 review abstracts for “lipoprotein metabolism”	8.75%	15.35%	20.98%
3,066 abstracts for “lipoprotein metabolism”	14.99%	38.25%	53.00%
50,000 abstracts containing “lipoprotein”			71.22%

## Discussion

The low coverage of the LMO in the data sets calls in question the document set selected and the suitability of the manually built LMO itself. The straightforward approach to select relevant documents from PubMed (review articles in “lipoprotein metabolism”) did not return enough documents to cover all of the LMO.

The LMO terms that were absent from the 50,000 PubMed abstracts were grouped in five categories: rarely occurring terms, rarely occurring variants of terms, very long terms, combinations of terms/variants and, finally, terms that should normally be easily found. Terms such as ‘experimentee’ (2) (absolute count of appearance in PubMed per term is given in parenthesis), ‘obesive’ (2), ‘test person’ (76) and ‘central fatness’ (9) are LMO terms, but rarely used by authors and, therefore, rarely appearing in PubMed. The second group contains variants of terms that appear rarely in PubMed, such as ‘Apo-F’ (14), ‘apolipoprotein c-3’ (4), ‘IDL I’ (1), ‘VLDL chol’ (34), ‘diabetis’ (37, instead of 270177 occurrences for ‘diabetes’), ‘free chol’ (0, instead of 2622 for ‘free cholesterol’), ‘hypolipoproteinaemia’ (5, “ae” spelling is rare), ‘insuline resistant’ (0, instead of 3912 for ‘insulin resistant’), ‘slo syndrome’ (36) and ‘sphingomyelinase deficiency disease’(0, MeSH synonym for ‘Niemann-Pick Disease’). However, we decided to include such terms in the LMO for completeness. The third category contains terms that are too long and, therefore, unlikely to appear as such in text: ‘receptor-mediated extra-hepatic cellular uptake’ (0), ‘macrophague cellular uptake’ (0), ‘predominance of large low-density lipoprotein particles’ (0) and ‘apob100 containing particles’ (2). However, given the initial purpose of the LMO for document retrieval, these terms were included to be recognized by the ontology-based text-mining methods [18]. The fourth group is a combination of the previous two, i.e. LMO terms that are long terms and contain rare variants of LMO terms, such as ‘elevated plasma-tg level’ (0), ‘increased total chol’ (0, instead of 116 for ‘increased total cholesterol’), ‘long-lived test person’ (0), ‘apoprotein b100 kinetics’ (0), ‘elevated plasma tg concentrations’ (0), and ‘decreased hdl-chol’ (4). The last group contains LMO terms that appear often in PubMed and should normally be identified, but are probably absent from the document set, due to its size or specificity. Such terms are ‘diabetes type I’ (126), ‘acetyl-coa c-acyltransferase’ (430), ‘apolipoprotein-c’ (1585), ‘type-II diabetic’ (1132), ‘long-lived population’ (23), ‘middle-aged adult’ (81), ‘human body composition’ (95), and ‘lipid poor HDL’ (12).

The third and fourth groups of terms belong to the same category as the hydrolase activity example described earlier. Composite terms like ‘receptor-mediated extra-hepatic cellular uptake’ and ‘predominance of large low-density lipoprotein particles’ could be easily broken into several semantic parts (e.g. receptor-mediated/ extra-hepatic/ cellular uptake, or more) and handled by an algorithm that could later compose them and still keep their semantics.

The terms that were predicted by most of the methods but were not in the LMO were further examined and grouped. These were either wrongly predicted ones, meaning phrases frequently occurring in the corpus, but not relevant to LMO, (~ 25% of the TFIDF predictions for the Top50 terms) (e.g. ‘review’, ‘type’, ‘article’, ‘role’, ‘event’, ‘use’) or vocabulary that could extend the current ontology (~ 40% of the TFIDF predictions for the Top50 terms). This would include disease-specific terms such as ‘atherosclerosis’, ‘cardiovascular risk’ and ‘atherogenic dyslipidemia’, drugs or other chemicals such as ‘statins’, ‘ezetimibe’ and ‘torcetrapib’, or even method and therapy related terms like ‘dose’ and ‘lipid lowering therapy’.

### Availability

The TFIDF term recognition is available as Web Service, described at 

## Conclusions

As pointed out in [3], automated term recognition is missing from many ontology design methologies. In this paper, we manually created an ontology for lipid metabolism with 223 concepts and 623 additional synonyms (846 terms in total), we derived design principles and systematically evaluated four methods for automated term recognition.

Automated predictions of up to 1000 terms generate in the order of 40-50% useful terms. Considering only the top 50 terms, the results improve up to 89% *average precision* for LMO + domain expert (defined earlier). This suggests that Automatic Term Recognition (ATR) methods can aid and speed up the process of ontology design by providing lists of useful domain-specific terms, but that they cannot (yet) replace the manually designed term lists. The key problem to further improve these results are composite terms which do not appear literally in text, like GO's ‘hydrolase activity, acting on ester bonds’ or LMO's ‘receptor-mediated extra-hepatic cellular uptake’.

Overall, our results show that ontology development can be performed in a semi-automatic way. The domain expert must have as initial input the output from an automatic term recognition method and proceed with enriching the ontology. The experiment as described aims at providing restrictions as well as decision points for including, excluding and reforming ontology terms. Once the domain expert acquires the list of candidate terms, he/she needs to decide on the relations between them. Formulation of questions is one of the most important steps in the ontology design process, helping to step from a list to an ontology.

We discussed principles for development of an ontology with text-mining as intended use, based on our personal experience from the manual development of the Lipoprotein Metabolism Ontology and GoPubMed. We related these principles to the performance of four different ATR methods and their agreement with the manually built LMO. Open problems relate to the choice of suitable text bodies for term recognition as well as generation of composite terms from basic ones.

## List of abbreviations used

LMO – Lipoprotein Metabolism Ontology

ATR – Automatic Term Recognition

OBO foundry – Open Biomedical Ontologies Foundry

MeSH – Medical Subject Headings

GO – Gene Ontology

OWL – Web Ontology Language

OWL-DL – Web Ontology Language-Description Logic

PMID – PubMed identifier

TFIDF – Term Frequency Inverse Document Frequency

RelFreq – Relative Frequency

NCBI – National Center for Biotechnology Information

NCI – National Cancer Institute

LDL – Low density lipoprotein

IDL – Intermediate density lipoprotein

HDL – High density lipoprotein

VLDL chol – Very low density lipoprotein cholesterol

ApoF – apolipoprotein F

## Competing interests

The authors declare that they have no competing interests.

## Authors' contributions

DA and LP developed the LMO, DA conceived the design principles and did the manual evaluation. TW implemented the TFIDF and RelFreq methods and automated the data collection and analysis. MS and CE supervised and coordinated the project. All authors have read and accepted the final manuscript.

## References

[B1] Soldatova LN, King RD (2005). Are the current ontologies in biology good ontologies?. Nat Biotechnol.

[B2] Sowa JF (2000). Knowledge Representation: Logical, Philosophical, and Computational Foundations.

[B3] Castro AG, Rocca-Serra P, Stevens R, Taylor C, Nashar K, Ragan MA, Sansone SA (2006). The use of concept maps during knowledge elicitation in ontology development processes – the nutrigenomics use case. BMC Bioinformatics.

[B4] Eilbeck K, Lewis SE, Mungall CJ, Yandell M, Stein L, Durbin R, Ashburner M (2005). The Sequence Ontology: a tool for the unification of genome annotations. Genome Biol.

[B5] Whetzel PL, Parkinson H, Causton HC, Fan L, Fostel J, Fragoso G, Game L, Heiskanen M, Morrison N, Rocca-Serra P, Sansone SA, Taylor C, White J, Stoeckert CJ (2006). The MGED Ontology: a resource for semantics-based description of microarray experiments. Bioinformatics.

[B6] Ashburner M, Ball CA, Blake JA, Botstein D, Butler H, Cherry JM, Davis AP, Dolinski K, Dwight SS, Eppig JT (2000). Gene ontology: tool for the unification of biology. The Gene Ontology Consortium. Nat Genet.

[B7] Evsikov AV, de VWN, Peaston AE, Radford EE, Fancher KS, Chen FH, Blake JA, Bult CJ, Latham KE, Solter D (2004). Systems biology of the 2-cell mouse embryo. Cytogenet Genome Res.

[B8] Wroe CJ, Stevens RD, Goble CA, Ashburner M, Altman RB, Dunker AK, Hunter L, Jung TA, Klein TE (2003). A methodology to migrate the gene ontology to a description logic environment using DAML+OIL. Proceedings of the 8th Pacific Symposium on Biocomputing (PSB 2003).

[B9] Rector AL, Rogers JE, Pole P, Brender J, Christensen JP, Scherrer JR and McNair P (1996). The GALEN high level ontology. Proceedings of Medical Informatics Europe '96 (MIE'96); Copenhagen.

[B10] Spackman KA (2004). SNOMED CT milestones: endorsements are added to already-impressive standards credentials. Healthc Inform.

[B11] Rosse C, Mejino JVL (2003). A reference ontology for biomedical informatics: the Foundational Model of Anatomy. J Biomed Inform.

[B12] Bodenreider O (2004). The Unified Medical Language System (UMLS): integrating biomedical terminology. Nucleic Acids Res.

[B13] Open Biomedical Ontologies Foundry. http://obofoundry.org/.

[B14] Bard J, Rhee SY, Ashburner M (2005). An ontology for cell types. Genome Biol.

[B15] Bodenreider O, Stevens R (2006). Bio-ontologies: current trends and future directions. Brief Bioinform.

[B16] Blake JA, Bult CJ (2006). Beyond the data deluge: data integration and bio-ontologies. J Biomed Inform.

[B17] Baker PG, Goble CA, Bechhofer S, Paton NW, Stevens R, Brass A (1999). An ontology for bioinformatics applications. Bioinformatics.

[B18] Doms A, Schroeder M (2005). GoPubMed: exploring PubMed with the Gene Ontology. Nucleic Acids Res.

[B19] GoPubMed. http://gopubmed.org/.

[B20] Nelson SJ, Johnston D, Humphreys BL, Bean CA, Green R (2001). Relationships in Medical Subject Headings. Relationships in the organization of knowledge.

[B21] Mueller HM, Kenny EE, Sternberg PW (2004). Textpresso: An Ontology-Based Information Retrieval and Extraction System for Biological Literature. PLoS Biol.

[B22] Perez-Iratxeta C, Pérez AJ, Bork P, Andrade MA (2003). Update on XplorMed: A web server for exploring scientific literature. Nucleic Acids Res.

[B23] GoMiner. http://discover.nci.nih.gov/gominer/.

[B24] Uschold M (1996). Building Ontologies: Towards a Unified Methodology. 16th Annual Conf of British Computer Society Specialist Group on Expert Systems: 1996.

[B25] NCI Cancer Nutrition Ontology Project. http://gforge.nci.nih.gov/projects/nutrition/.

[B26] Automatic labeling of document clusters. http://www.cis.upenn.edu/~popescul/Publications/popescul00labeling.pdf.

[B27] Protégé OWL plug-in. http://protege.stanford.edu/overview/protege-owl.html.

[B28] CmapTools. http://cmap.ihmc.us/.

[B29] Smith B, Köhler J, Kumar A, Rahm E (2004). On the Application of Formal Principles to Life Science Data: a Case Study in the Gene Ontology. Proceedings of the First International Workshop on Data Integration in the Life Sciences, DILS.

[B30] Aranguren ME, Bechhofer S, Lord P, Sattler U, Stevens R (2007). Understanding and using the meaning of statements in a bio-ontology: recasting the Gene Ontology in OWL. BMC Bioinformatics.

[B31] Definition of mammals. http://www.biology-online.org/dictionary/Mammals.

[B32] Hoehndorf R, Loebe F, Kelso J, Herre H (2007). Representing default knowledge in biomedical ontologies: Application to the integration of anatomy and phenotype ontologies. BMC Bioinformatics.

[B33] Rector A, Altman RB, Dunker AK, Hunter L, Jung TA, Klein TE (2004). Defaults, context, and knowledge: alternatives for OWL-indexed knowledge bases. Proceedings of the 9th Pacific Symposium on Biocomputing (PSB 2004).

[B34] Ogren PV, Cohen KB, Acquaah-Mensah GK, Eberlein J, Hunter L, Altman RB, Dunker AK, Hunter L, Jung TA, Klein TE (2004). The compositional structure of Gene Ontology terms. Proceedings of the 9th Pacific Symposium on Biocomputing (PSB 2004).

[B35] Ogren PV, Cohen KB, Hunter L, Altman RB, Dunker AK, Hunter L, Jung TA, Klein TE (2005). Implications of compositionality in the Gene Ontology for its curation and usage. Proceedings of the 10th Pacific Symposium on Biocomputing (PSB 2005).

[B36] Stefanowski J, Weiss D, Ruiz EM, Segovia J, Szczepaniak PS (2003). Carrot^2^ and language properties in Web search results clustering. Proceedings of the First International Atlantic Web Intelligence Conference.

[B37] Lame G (2004). Using NLP techniques to identify legal ontology components: Concepts and Relations. Artificial Intelligence and Law.

[B38] Berneis K, Rizzo M (2004). LDL size: does it matter?. Swiss Med Wkly.

[B39] McCray A, Srinivasan S, Browne A (1994). Lexical methods for managing variation in biomedical terminologies. Proceedings of the 18th Annual Symposium on Computer Applications in Medical Care.

[B40] Agirre E, Stevenson M, Agirre E, Edmonds P Knowledge Sources for WSD. Word Sense Disambiguation: Algorithms and Applications.

[B41] Cimiano P, Völker J, Montoyo A, Muñoz R, Métais E: Springer (2005). Text2Onto - A Framework for Ontology Learning and Data-driven Change Discovery. Proceedings of the 10th International Conference on Applications of Natural Language to Information Systems (NLDB 2005).

[B42] Navigli R, Verlardi P (2004). Learning Domain Ontologies from Document Warehouses and Dedicated Websites. Computational Linguistics.

[B43] Frantzi K, Ananiadou S, Mima H (2000). Automatic recognition of multi-word terms: the C-value/NC-value method. International Journal on Digital Libraries.

[B44] Fellbaum C (1998). WordNet: an electronic lexical database.

[B45] Hearst M (1992). Automatic acquisition of hyponyms from large text corpora. Proceedings of the Fourteenth International Conference on Computational Linguistics.

